# Performance of Guideline‐Recommended Approaches to Echocardiographic Investigation for Pulmonary Hypertension: Analysis of the CIPHER Study

**DOI:** 10.1002/pul2.70258

**Published:** 2026-02-17

**Authors:** Luke S. Howard, David G. Kiely, Allan Lawrie, Bradley A. Maron, Ioana R. Preston, Stephan Rosenkranz, Mark Toshner, Martin R. Wilkins, Yiu‐Lian Fong, Debbie Quinn, Dimitri Stamatiadis, Matthieu Villeneuve, Kelly M. Chin

**Affiliations:** ^1^ National Pulmonary Hypertension Service, Hammersmith Hospital London UK; ^2^ Sheffield Pulmonary Vascular Disease Unit and NIHR Biomedical Research Centre, Royal Hallamshire Hospital, Sheffield Teaching Hospitals NHS Foundation Trust Sheffield UK; ^3^ National Heart and Lung Institute, Imperial College London London UK; ^4^ Department of Medicine University of Maryland School of Medicine Baltimore Maryland USA; ^5^ University of Maryland‐Institute for Health Computing Bethesda Maryland USA; ^6^ Department of Pulmonary and Critical Care Medicine Lahey Hospital and Medical Center Burlington Massachusetts USA; ^7^ Department of Cardiology and Cologne Cardiovascular Research Center (CCRC), Heart Center University Hospital Cologne Cologne Germany; ^8^ Department of Medicine University of Cambridge Cambridge UK; ^9^ Johnson & Johnson New Brunswick New Jersey USA; ^10^ Johnson & Johnson Allschwil Switzerland; ^11^ Division of Pulmonary and Critical Care Medicine UT Southwestern Medical Center Dallas Texas USA

**Keywords:** diagnosis, diagnostic, echocardiography, screening, transthoracic echocardiogram

## Abstract

Guidelines recommend different approaches to investigate for pulmonary hypertension (PH) by transthoracic echocardiography (TTE). We used data from the CIPHER study (NCT04193046) to prospectively evaluate TTE detection of PH. Participants newly referred to PH clinics who underwent right heart catheterization (RHC) within 6 weeks and TTE within 60 days of enrolment (blinded central TTE reading) were classified by TTE probability of PH applying (i) the 2015 European Society of Cardiology (ESC)/European Respiratory Society (ERS) TTE algorithm or (ii) right ventricular systolic pressure (RVSP) > 40 mmHg. For calculation of sensitivity and specificity, ‘non‐assessable’ patients (peak tricuspid regurgitation velocity [TRV] missing or ≤ 2.8 m/s with missing data on other echocardiographic signs) and patients with missing RVSP were counted as PH‐negative. Performance was measured against RHC‐confirmed diagnosis of mean pulmonary artery pressure > 20 mmHg. Of 475 patients included, 345 (73%) had PH. Using the ESC/ERS algorithm, PH probability was high, intermediate, low and non‐assessable for 198, 104, 22 and 151 patients and PH prevalence was 98%, 75%, 23%, and 44%, respectively. Seventy‐three patients were missing RVSP and 292 had RVSP > 40 mmHg. The two TTE approaches achieved similar results: sensitivity was 79%–77%, specificity was 78%–79%. This prospective study of patients newly referred to PH centres for RHC found similar sensitivity and specificity when using either RVSP > 40 mmHg or the 2015 ESC/ERS TTE algorithm. Among patients who were low‐probability or non‐assessable by ESC/ERS algorithm, 42% had PH, highlighting the persistent need for additional non‐invasive investigative tools.

## 1 Introduction

Pulmonary hypertension (PH) is a progressive, life‐threatening condition characterized by elevated mean pulmonary artery pressure (mPAP) [1]. The mPAP threshold used to define PH was recently lowered from ≥ 25 mmHg (2015 European Society of Cardiology [ESC]/European Respiratory Society [ERS] guidelines on the management of PH) [2] to > 20 mmHg (2022 ESC/ERS guidelines) [1]. PH diagnosis must be confirmed by right heart catheterization (RHC); however, patients often wait over a year for diagnosis [1].

According to the latest ESC/ERS guidelines, initial assessment of unexplained dyspnoea should include medical history, physical exam, N‐terminal pro‐brain natriuretic peptide (NT‐proBNP) levels and resting electrocardiogram [1]. In cases of suspected PH, a transthoracic echocardiogram (TTE) is recommended to further determine the probability of PH, before considering RHC [1]. There have been two main approaches to investigating PH by TTE. The ESC/ERS guidelines recommend classifying patients as low, intermediate or high probability of PH according to their peak TRV measurement (≤ 2.8 m/s or not measurable; 2.9–3.4 m/s; > 3.4 m/s, respectively), and whether they have other echocardiographic signs of PH. These other echocardiographic signs are divided into three categories (A, the ventricles; B, the pulmonary artery; C, the inferior vena cava and right atrium). Signs from at least two categories must be present to shift a patient's probability of PH to intermediate (if TRV ≤ 2.8 m/s or not measurable) or high (if TRV 2.9–3.4 m/s) [1, 2]. In contrast, the 2010 American Society of Echocardiography (ASE) guidelines focus on estimated right ventricular systolic pressure (RVSP), recommending that patients with RVSP > 40 mmHg be further evaluated for suspected PH [3]. However, the 2025 update of these guidelines now provide an algorithm more similar to that of the ESC/ERS guidelines, recommending further evaluation of patients with peak TRV ≥ 2.9 m/s or ≥ 2.8 m/s with adjunctive signs of PH [4].

Only a few, mostly retrospective, studies have examined the performance of the ESC/ERS recommended parameters [5, 6, 7, 8], and not all participants had a RHC‐confirmed diagnosis. The contribution of NT‐proBNP to PH detection has mostly been studied in specific PH subgroups [9, 10, 11, 12]. We used data from the prospective, multicentre study for the identification of biomarker signatures for early detection of PH (CIPHER study, NCT04193046) to examine the real‐world performance of the 2015 ESC/ERS recommended parameters (alone and in combination), RVSP and NT‐proBNP in identifying PH against the RHC‐confirmed diagnosis.

## 2 Methods

### 2.1 Objective of Analysis

CIPHER was a prospective, non‐therapeutic, multicentre study that primarily aimed to develop a micro‐ribonucleic acid (miRNA)‐based signature (miRNA only or miRNA and NT‐proBNP) for detecting PH and estimating its performance along with that of TTE in detecting PH, as measured against RHC. The primary results have been published [13]. The TTE results included the performance of TRV > 2.4 m/s alone or in combination with measures recommended in the 2015 ESC/ERS guidelines, excluding patients whose TTE results were inadequate to provide a PH probability. To gain a better understanding of the real‐world performance of TTE detection of PH, this analysis included all patients undergoing echocardiogram (*N* = 475), including 28 patients excluded from the earlier report due to missing data. Additional analyses performed include assessment of the sensitivity and specificity of TRV and RVSP in detecting RHC‐confirmed PH at different cutpoints, and the performance of individual guideline‐recommended echocardiographic measures in detecting RHC‐confirmed PH, assessed using likelihood ratios. This analysis also assessed the potential of NT‐proBNP as a diagnostic biomarker in PH.

### 2.2 Study Design and Participants

The CIPHER study design has been described previously [13]. This analysis includes patients who were newly referred to specialist PH clinics (Supporting Table 1) and were evaluated by RHC within 6 weeks and a TTE within 60 days either side of enrolment.

All World Health Organization (WHO) PH groups were represented: any cases where the PH group had not been classified or did not conform to the definitions in Supplemental Table 2 were submitted to a disease classification adjudication committee (comprised of authors L.H, D.G.K, A.L, B.A.M, I.R.P, S.R, M.T, M.R.W, and K.M.C) as described in Lawrie et al. [13].

Blood samples were taken at enrolment and the analysis of NT‐proBNP was performed at a central laboratory. All TTEs were centrally read in a blinded manner. A TTE that was already performed before enrolment could be used if there had been no change in the clinical status or treatment (PH therapies) of the participant and providing the TTE was performed according to local guidelines and could be de‐identified and centrally read (i.e., its images could be loaded to the central website). TTE operators were not given any instructions by study staff with respect to how to conduct the TTEs, as the intention was to understand real‐world performance.

### 2.3 Classification of Patients by the 2015 ESC/ERS TTE Algorithm

**Figure 1:**
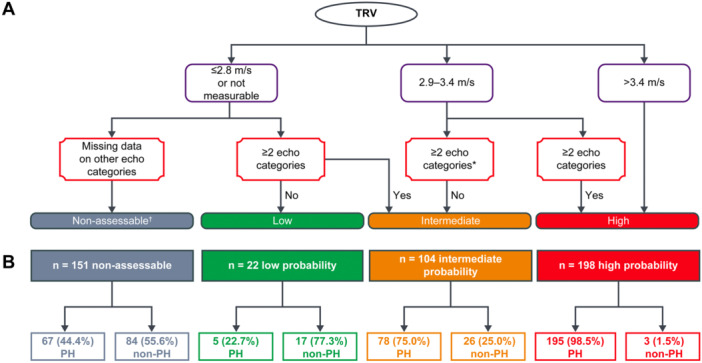
Patient flowchart showing (A) how patients were classified according to echocardiographic findings based on the 2015 ESC/ERS guidelines on PH management [1, 2] and (B) how many CIPHER patients were in each of these TTE‐defined groups and how many of each group had PH. *A patient with TRV 2.9–3.4 m/s and missing data on other echocardiographic signs of PH was classed as having intermediate probability of PH. †CIPHER‐specific definition, not used in the ESC/ERS guidelines. PH is defined as mean pulmonary artery pressure > 20 mmHg. Figure adapted from Figure 1 of Slegg et al. [8]. Echo Res Pract 2022; doi: 10.1186/s44156‐022‐00010‐9; Creative Commons Attribution 4.0 International License. Category A signs of PH (i.e., signs in the ventricles) as right ventricle/left ventricle basal diameter ratio > 1.0, flattening of the interventricular septum (left ventricular eccentricity index > 1.1 in systole and/or diastole); Category B signs of PH (i.e. signs in the pulmonary artery) as right ventricular outflow Doppler acceleration time < 105 msec and/or midsystolic notching, early diastolic pulmonary regurgitation velocity > 2.2 m/sec, pulmonary artery diameter > 25 mm; Category C signs of PH (i.e., signs in the inferior vena cava and right atrium) as inferior cava diameter > 21 mm with decreased inspiratory collapse ( < 50% with a sniff or < 20% with quiet inspiration), right atrial area (end‐systole) > 18 cm^2^. ERS, European Respiratory Society; ESC, European Society of Cardiology; PH, pulmonary hypertension; TRV, tricuspid regurgitation velocity; TTE, transthoracic echocardiogram.

To assess the performance of the 2015 ESC/ERS TTE algorithm, all patients were classified by their probability of PH according to the 2015 guideline recommendations (Figure 1A; Supplemental Appendix 1) [2]. The ESC/ERS guidelines do not advise on how to classify patients when information on the other echocardiographic signs of PH (categories A–C) is missing [2]. In this analysis, patients with low ( ≤ 2.8 m/s) or missing peak TRV were classified as intermediate probability of PH if they had confirmed echocardiographic signs of PH in two or more of the 2015 ERS/ESC categories (Supplemental Figure 1A) and as low probability if such signs could be ruled‐out based on the available measures (Supplemental Figure 1B). The remainder of patients with low or missing TRV were classified as non‐assessable (Supplemental Figure 1C).

### 2.4 Study Analyses

This analysis measured the sensitivity, specificity, positive predictive value (PPV) and negative predictive value (NPV) of the 2015 ESC/ERS TTE guidelines. Patients with a high or intermediate probability of PH were considered PH‐positive by TTE, and non‐assessable patients were grouped with low probability patients as PH‐negative by TTE. The performance of peak TRV measurement alone (with TRV ≥ 2.9 m/s considered PH‐positive, as per ESC/ERS guidelines) [2] and RVSP alone (with RVSP > 40 mmHg [3] considered PH‐positive) was also assessed. Patients with missing RVSP/TRV measurements were grouped with low RVSP/TRV measurements as PH‐negative. RVSP was calculated as ‘4 x peak TRV^2^ + inferior vena cava (IVC) diameter’ or ‘4 x peak TRV^2^ + 10 (if IVC diameter missing)’.

This analysis also measured the sensitivity and specificity of TRV alone and the full 2015 ESC/ERS TTE algorithm in detecting pre‐capillary PH, using the 6th World Symposium on PH (WSPH) definition (mPAP > 20 mmHg and PVR ≥ 3 WU) [14], which excludes isolated post‐capillary PH.

We computed from our data optimal cut‐points of TRV, RVSP and NT‐proBNP for distinguishing PH from non‐PH. Receiver operating characteristic (ROC) curves were generated for each of these parameters and the thresholds for PH were identified using the Youden index (J) method (i.e., maximum value of Youden's index sensitivity + specificity −1) [15] and the F1 method (maximum of the harmonic average of sensitivity and PPV) [16]. Patients missing a measurement were excluded for determination of the optimal cut‐points, whereas performance of the cut‐points was assessed both when these patients were included (as PH‐negative) or excluded.

The diagnostic value of NT‐proBNP measurements was also assessed in the overall cohort and in non‐assessable and low probability patients. This subgroup analysis was done to understand whether NT‐proBNP measurements could help guide next steps in scenarios where patients with PH might otherwise be overlooked for RHC. Patients with missing NT‐proBNP measurements were excluded from this analysis.

All of the above analyses used the RHC reading as the confirmatory diagnosis: the mPAP threshold of > 20 mmHg was used as a main analysis and a supplemental analysis used mPAP ≥ 25 mmHg. Hereafter, the term PH will refer to mPAP > 20 mmHg and non‐PH will indicate mPAP ≤ 20 mmHg, unless otherwise specified.

We evaluated the individual contribution of each of the seven echocardiographic parameters that the 2015 ESC/ERS guidelines recommend measuring in addition to TRV, by calculating likelihood ratios (LR + ) and using logistic regression (details in Supplemental Appendix 2).

### 2.5 Statistical Analysis

All analyses except the logistic regression analysis of the seven echocardiographic parameters were descriptive only (Supplemental Appendix 3).

### 2.6 Ethical Approval

The protocol and other study documentation for each study were reviewed and approved by the relevant Institutional Review Board/Independent Ethics Committee for each site before the studies were initiated (detailed in Lawrie et al. [13]) and each patient gave written informed consent.

## 3 Results

### 3.1 Patients

In total, 475 patients in the CIPHER study met the inclusion criteria for this analysis. All patients were newly referred to a PH centre and underwent TTE within 60 days and RHC within 6 weeks of enrolment. Fifty‐eight percent of patients were female and the mean age was 61.7 (14.4) years. Median (Q1, Q3) time between RHC and TTE was –1 (–5, 1) days. TTEs performed before enrolment could be included if they met eligibility criteria: of 475 TTEs, 284 were performed before enrolment (5 of those more than 60 days before enrolment), 116 were performed on the day of enrolment and 75 occurred after enrolment (4 more than 60 days after enrolment). PH was identified at RHC in 345 (73%) using an mPAP threshold of > 20 mmHg and all World Health Organization (WHO) groups of PH were included (Table 1).

**Table 1:** Baseline characteristics of patients by their TTE probability of PH.

Characteristic	Total population (*n* = 475)	TTE probability of PH			
		High (*n* = 198)	Intermediate (*n* = 104)	Low (*n* = 22)	Non‐assessable (*n* = 151)
Age, years	61.7 ± 14.4	63.8 ± 14.2	64.8 ± 12.5	48.4 ± 15.5	58.9 ± 14.1
Female, %	57.7	53.0	55.8	77.3	62.3
mPAP, mmHg	32.6 ± 14.9	44.8 ± 11.5	28.4 ± 10.5	18.2 ± 4.8	21.4 ± 9.1
PAWP, mmHg	10.9 ± 5.3	10.8 ± 4.9	12.4 ± 6.2	9.4 ± 4.0	10.2 ± 5.3
PVR, WU	5.8 ± 5.5	9.7 ± 5.6	4.0 ± 4.1	1.8 ± 1.5	2.4 ± 2.0
CO, L/min	4.9 ± 1.8	4.3 ± 1.9	5.0 ± 2.0	5.4 ± 1.3	5.5 ± 1.5
NT‐proBNP, ng/L	1321.5 ± 2000.0	2280.8 ± 2316.8	961.8 ± 1476.5	93.6 ± 60.7	461.7 ± 1328.4
PH Group					
Group 1 PH	117 (24.6%)	85 (42.9%)	17 (16.4%)	0	15 (9.9%)
Group 2 PH	60 (12.6%)	22 (11.1%)	24 (23.1%)	1 (4.6%)	13 (8.6%)
Group 3 PH	40 (8.4%)	25 (12.6%)	11 (10.6%)	0	4 (2.7%)
Group 4 PH	65 (13.7%)	47 (23.7%)	9 (8.7%)	2 (9.1%)	7 (4.6%)
Group 5 PH	13 (2.7%)	7 (3.5%)	4 (3.9%)	1 (4.6%)	1 (0.7%)
Unclear	11 (2.3%)	4 (2.0%)	1 (1.0%)	0	6 (4.0%)
Unclassifiable	39 (8.2%)	5 (2.5%)	12 (11.5%)	1 (4.6%)	21 (13.9%)
No PH	130 (27.4%)	3 (1.5%)	26 (25.0%)	17 (77.3%)	84 (55.6%)
**BMI, kg/m** ^**2**^	29.3 ± 7.5	29.0 ± 6.5	27.9 ± 6.1	27.6 ± 7.4	30.9 ± 9.1
**Race**	*n* = 469	*n* = 196	*n* = 102	*n* = 22	*n* = 149
American Indian or Alaskan Native	3 (0.6%)	1 (0.5%)	2 (2.0%)	0	0
Asian	6 (1.3%)	3 (1.5%)	1 (1.0%)	1 (4.6%)	1 (0.7%)
Black or African	12 (2.6%)	7 (3.6%)	0	1 (4.6%)	4 (2.7%)
American Native					
Native Hawaiian or other Pacific Islander	1 (0.2%)	1 (0.5%)	0	0	0
White	431 (91.9%)	176 (89.8%)	99 (97.1%)	17 (77.3%)	139 (93.3%)
Not reported	15 (3.2%)	7 (3.6%)	0	3 (13.6%)	5 (3.4%)
Multiple	1 (0.2%)	1 (0.5%)	0	0	0
**WHO FC**	*n* = 357	*n* = 194	*n* = 78	*n* = 7	*n* = 78
I	10 (2.8%)	3 (1.6%)	3 (3.9%)	1 (14.3%)	3 (3.9%)
II	86 (24.1%)	34 (17.5%)	24 (30.8%)	3 (42.9%)	25 (32.1%)
III	236 (66.1%)	136 (70.1%)	48 (61.5%)	3 (42.9%)	49 (62.8.%)
IV	25 (7.0%)	21 (10.8%)	3 (3.9%)	0	1 (1.3%)

*Note:* Values represent mean ± SD for continuous variables and *n* (%) for categorical variables.

Abbreviations: CO, cardiac output; FC, functional class; mPAP, mean pulmonary artery pressure; NT‐proBNP, N‐terminal pro‐brain natriuretic peptide; PAWP, pulmonary artery wedge pressure; PVR, pulmonary vascular resistance; TTE, transthoracic echocardiogram; WHO, World Health Organization; WU, wood units.

### 3.2 Performance of the 2015 ESC/ERS Guidelines on TTE Evaluation

Using the 2015 ESC/ERS TTE algorithm, the echocardiographic probability of PH was considered high, intermediate and low for 198, 104, and 22 patients, respectively, while 151 patients had non‐assessable echocardiograms (Table 1 and Figure 1B). Non‐assessable TTEs were most often missing conclusive information on category B parameters (47.6% TTEs), compared with categories C (40.1%) and A (32.0%). The most common reason for missing TTE data was inadequate TTE views obtained (Supplemental Table 4; echocardiograms were performed locally and read centrally).

The performance of the full 2015 ESC/ERS TTE algorithm is shown in Table 2. The prevalence of PH in the individual TTE‐defined groups was 98% (high), 75% (intermediate), 23% (low) and 44% (non‐assessable) (Figure 1B). Sensitivity and specificity were 79% and 78%, respectively. PPVs and NPVs are shown in Table 2; however, PPV is directly proportional to disease prevalence, which is naturally high (73%) in this study population.

**Table 2:** Performance of echocardiographic parameters in distinguishing PH (mPAP > 20 mmHg) from non‐PH (mPAP ≤ 20 mmHg).

TTE classification	RHC diagnosis of PH		Prevalence of PH by RHC	Sensitivity^a^	Specificity^a^	PPV	NPV
	Yes	No		[95% CI]	[95% CI]	[95% CI]	[95% CI]
**Performance of full 2015 ESC/ERS TTE algorithm**							
High	195	3	98.5%	79.1%		90.4%	
Intermediate	78	26	75.0%	[74.5, 83.1]		[86.5, 93.2]	
Low	5	17	22.7%		77.7%		58.4%
Non‐assessable	67	84	44.4%		[69.8, 84.0]		[50.9, 65.5]
**Performance of peak TRV alone – ESC/ERS PH guideline cutoffs**							
High ( > 3.4 m/s)	145	2	98.6%	69.0%		93.3%	
Intermediate (2.9–3.4 m/s)	93	15	86.1%	[63.9, 73.6]		[89.6, 95.8]	
Low ( ≤ 2.8 m/s)	74	73	50.3%		86.9%		51.4%
TRV missing	33	40	45.2%		[80.1, 91.7]		[44.8, 57.9]
**Performance of RVSP alone – RVSP** > **40 mmHg as cutoff**							
RVSP > 40 mmHg	264	28	90.4%	76.5%		90.4%	
				[71.8, 80.7]		[86.5, 93.3]	
RVSP ≤ 40 mmHg	48	62	43.6%		78.5%		55.7%
RVSP missing	33	40	45.2%		[70.6, 84.7]		[48.5, 62.7]

Abbreviations: CI, confidence interval; ERS, European Respiratory Society; ESC, European Society of Cardiology; mPAP, mean pulmonary artery pressure; NPV, negative predictive value; PPV, positive predictive value; PH, pulmonary hypertension; RHC, right heart catheterization; RVSP, right ventricular systolic pressure; TRV, tricuspid regurgitation velocity; TTE, transthoracic echocardiogram.

^a^ For these calculations, patients who were non‐assessable (by ESC/ERS guidelines) or had missing TRV/RVSP measurements were grouped with low probability patients as being PH‐negative by TTE.

### 3.3 Performance of TRV or RVSP Alone

**Figure 2:**
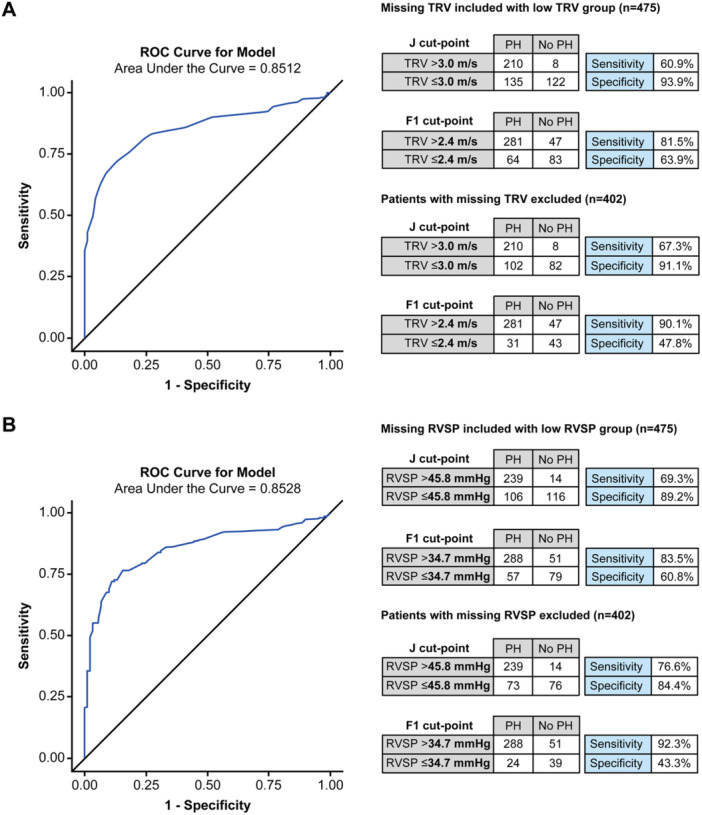
ROC curves and performance of data‐driven cut‐points for (A) TRV and (B) RVSP, in distinguishing PH (mPAP > 20 mmHg) from non‐PH (mPAP ≤ 20 mmHg), based on overall population. Performance is shown for the following scenarios: (i) when patients with missing values are included in the group with patients who have a low measurement and, (ii) when patients with missing values are excluded from the calculations of sensitivity and specificity. The thresholds for PH were identified using (i) the Youden index (J) method (i.e., maximum value of Youden's index sensitivity + specificity −1) and (ii) the F1 method (maximum of the harmonic average of sensitivity and PPV). mPAP, mean pulmonary artery pressure; PH, pulmonary hypertension; PPV, positive predictive value; ROC, receiver operating characteristic; RVSP, right ventricular systolic pressure; TRV, tricuspid regurgitation velocity.

TRV/RVSP measurements were missing for 73 (15%) patients. Using RVSP > 40 mmHg [3], and considering missing/non‐measurable RVSP as PH‐negative, resulted in a similar performance as the full 2015 ESC/ERS guidelines [2]: sensitivity was 77% and specificity was 79% (Table 2). In contrast, using peak TRV ≥ 2.9 m/s (ESC/ERS cut‐off [2]) had lower sensitivity (69%) and higher specificity (87%). Alternative cut‐points based on the ROC curves for TRV and RVSP (Figure 2) show a trade‐off in sensitivity versus specificity.

### 3.4 Contribution of Each of the Seven Other Echocardiographic Parameters

We assessed the performance of each of the seven non‐TRV echocardiographic parameters recommended by the 2015 ESC/ERS guidelines. Measures with the highest LR+ included left ventricle (LV) eccentricity index, right ventricle (RV)/LV basal diameter, mid‐systolic notching and IVC diameter, with an LR+ of at least 7.7 (Table 3). We also assessed the performance using logistic regression analyses. Missing values were included in the analysis and assumed to be either normal (Supplemental Table 5) or abnormal (Supplemental Table 6). Mid‐systolic notching, RV/LV basal diameter/area ratio > 1.0 and right atrial area > 18 cm^2^ were statistically significant predictors of PH, regardless of how missing values were dealt with (Supplemental Table 5 and Supplemental Table 6).

**Table 3:** The prevalence of PH among patients who have normal, abnormal and missing echocardiographic readings.

Parameter	Categorization of parameter	RHC‐confirmed diagnosis		Prevalence of PH, %	LR + (missing values considered normal)	LR + (missing values excluded)
		PH	Non‐PH			
RV/LV basal diameter ratio	Abnormal	162	4	97.6		
	Normal	130	85	60.5	15.3	12.3
	Missing	53	41	56.4		
LV eccentricity index	Abnormal	95	1	99.0		
	Normal	155	68	69.5	35.8	26.2
	Missing	95	61	60.9		
RV outflow doppler accelerator time	Abnormal	164	25	86.8		
	Normal	28	30	48.3	2.5	1.9
	Missing	153	75	67.1		
Mid‐systolic notching	Abnormal	109	3	97.3		
	Normal	66	49	57.4	13.7	10.8
	Missing	170	78	68.6		
Early diastolic pulmonary regurgitation velocity	Abnormal	10	0	100.0		
	Normal	75	10	88.2	N/A	N/A
	Missing	260	120	68.4		
Pulmonary artery diameter	Abnormal	130	23	85.0		
	Normal	56	28	66.7	2.1	1.5
	Missing	159	79	66.8		
Right atrial area	Abnormal	208	27	88.5		
	Normal	76	55	58.0	2.9	2.2
	Missing	61	48	56.0		
Inferior cava diameter	Abnormal	29	1	96.7		
	Normal	80	28	74.1	10.9	7.7
	Missing	236	101	70.0		

*Note:* Each parameter has two LR+ reported: one in which sensitivity and specificity were calculated based on missing values being grouped with normal, and another where missing values were grouped with abnormal values. LR + , likelihood ratios (Sensitivity/(1‐Specificity) ‐ probability that a positive test would be expected in a patient with PH divided by the probability that a positive test would be expected in a patient without PH. LR+, likelihood ratios; LV, left ventricle; N/A, not applicable; PH, pulmonary hypertension; RHC, right heart catheterization; RV, right ventricle.

### 3.5 Performance of NT‐proBNP in Evaluation of Suspected PH

**Figure 3:**
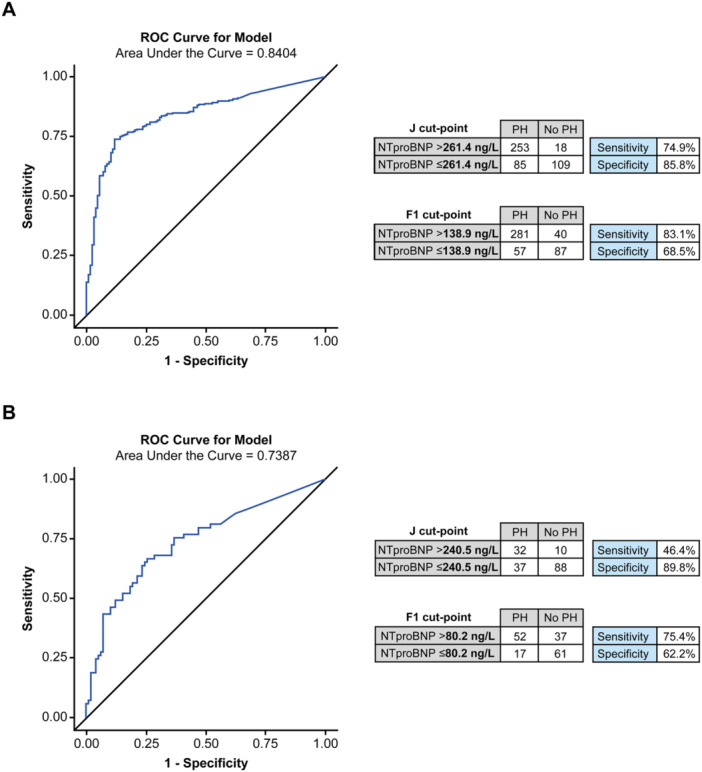
ROC curves and performance of data‐driven cut‐points of NT‐proBNP in distinguishing PH (mPAP > 20 mmHg) from non‐PH (mPAP ≤ 20 mmHg) in (A) the overall cohort, *n* = 465 and (B) patients who are non‐assessable by TTE or who have low echocardiographic probability of PH, *n* = 167. Patients with missing NT‐proBNP are excluded. The thresholds for PH were identified using (i) the Youden index (J) method (i.e., maximum value of Youden's index sensitivity + specificity −1) and (ii) the F1 method (maximum of the harmonic average of sensitivity and PPV). mPAP, mean pulmonary artery pressure; NT‐proBNP, N‐terminal pro‐brain natriuretic peptide; PH, pulmonary hypertension; PPV, positive predictive value; ROC, receiver operating characteristic; TTE, transthoracic echocardiogram.

Figure 3 summarizes the performance of NT‐proBNP. Applying our data‐driven NT‐proBNP cut‐offs (> 138.9 ng/L and > 261.4 ng/L) achieved reasonable sensitivity and specificity, and the area under curve (AUC) at 0.84 was just below that achieved by RVSP and TRV. In addition, in patients with a low or non‐assessable echocardiographic probability of PH, applying an NT‐proBNP threshold of > 80.2 ng/L (F1 cut‐point) identified 52 PH patients and achieved a sensitivity of 75% and a specificity of 62% for predicting PH in this group of 167 patients (Figure 3).

### 3.6 Results Using mPAP ≥ 25 mmHg as Definition of PH

We also performed analyses using the older mPAP cut‐off of ≥ 25 mmHg to define PH. PH was present in 296 (62%) of patients using this definition. Baseline characteristics of PH and non‐PH patients are shown in Supplemental Table 7. Sensitivity was higher for echocardiographic detection of mPAP ≥ 25 mmHg than mPAP > 20 mmHg, while the opposite trend was observed for specificity (Supplemental Table 8).

### 3.7 Results in Patients With Pre‐Capillary PH (6th WSPH Definition)

Baseline characteristics of patients using the 6th WSPH definition of pre‐capillary PH (mPAP > 20 mmHg and PVR ≥ 3 WU) [14] are shown in Supplementary Table 9. Overall, 263 (55%) of patients were identified as having pre‐capillary PH. Using the full 2015 ESC/ERS TTE algorithm for the detection of PH among patients with pre‐capillary PH resulted in a sensitivity of 87% and a specificity of 65%, whereas the sensitivity and specificity of using TRV ≥ 2.9 m/s was 80% and 79%, respectively (Supplementary Table 10).

## 4 Discussion

**Figure 4:**
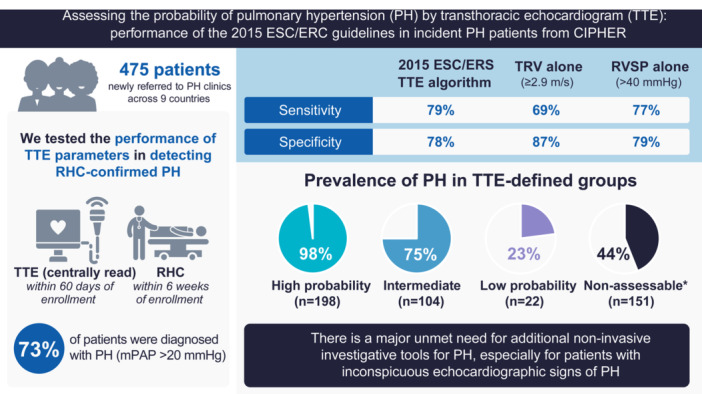
Visual summary of key methods and results. *‘Non‐assessable’ defined as patients with peak TRV missing or ≤ 2.8 m/s with missing data on other echocardiographic signs of PH. For sensitivity and specificity calculations, low probability and non‐assessable patients were considered PH‐negative. ESC, European Society of Cardiology; ERS, European Respiratory Society; mPAP, mean pulmonary artery pressure; PH, pulmonary hypertension; RHC, right heart catheterization; RVSP, right ventricular systolic pressure; TRV, tricuspid regurgitation velocity; TTE, transthoracic echocardiogram.

This prospective study of patients newly referred to PH centres for RHC evaluated the performance of the 2015 ESC/ERS guideline recommendations for TTE investigation for PH, compared with using RVSP, TRV or NT‐proBNP alone (Figure 4). Sensitivity was higher for identifying PH using the full algorithm (i.e., TRV and presence/absence of other echocardiographic signs) versus using the ESC/ERS threshold for TRV alone (79% vs. 69%), while specificity was higher when using TRV alone (87% vs. 78%). Notably, using RVSP > 40 mmHg [3] achieved a sensitivity and specificity similar to those achieved by the full ESC/ERS guidelines (sensitivity: 77% vs. 79%, specificity: 79% vs. 78%).

### 4.1 Performance of RVSP or TRV Alone to Detect PH

The current ESC/ERS guidelines recommend using TRV, rather than RVSP (TRV plus estimated right atrial pressure), due to the risk of amplification errors with derived variables [1]. In our study, both RVSP and TRV alone performed reasonably well, and with similar overall performance (AUC of 0.851 for TRV and 0.853 for RVSP). These values are consistent with those from a meta‐analysis of 27 studies (*n* = 4386) on the echocardiographic detection of PH that reported a pooled AUC of 0.88 for RVSP [17], and with recent studies that found an AUC of 0.87–0.91 for TRV [5, 7, 8].

Sensitivity and specificity of TRV/RVSP varied depending on the cut‐off used, with RVSP > 40 mmHg [3] outperforming TRV ≥ 2.9 m/s [1] in terms of sensitivity (77% vs. 69%) at a cost of lower specificity. The greater sensitivity achieved with RVSP > 40 mmHg (compared with TRV ≥ 2.9 m/s) is consistent with the TTE‐estimated right atrial pressures, which were relatively high (and higher than equivalent RHC‐measured right atrial pressures) (Supplemental Table 7). The trade‐off between sensitivity and specificity was observed for both measures in general, with lower cut‐points resulting in a higher sensitivity and lower specificity than higher cut‐points. Since PH is life‐threatening, yet often treatable, the cut‐points should be selected based on optimizing for sensitivity. This may also vary depending on the patient population. For example, given our findings, a lower threshold of RVSP > 35 mmHg may be applied to patients with suspected PH, at the risk of more false positives.

Even using cut‐offs optimized for sensitivity and PPV, up to around one‐fifth of PH patients would have been missed in the current study if using only TRV or RVSP. This occurs because TRV was not measurable in 15% of TTEs, consistent with prior reports of TRV/RVSP being missing from 15%–45% of TTEs [6, 8, 18]. Common reasons for missing values include inadequate tricuspid regurgitation itself, as well as difficulties in obtaining TTE views due to obesity or other factors [19, 20].

### 4.2 Performance of the 2015 ESC/ERS TTE Algorithm

The 2015 ESC/ERS TTE algorithm achieved a slightly better sensitivity than RVSP > 40 mmHg and TRV ≥ 2.9 m/s, picking up an additional nine and 35 patients, respectively. However, overall, there was no substantial benefit in applying the full guidelines versus TRV or RVSP alone. A retrospective single‐centre study by D'Alto et al. (*n* = 278 patients with TTE and RHC data) also found the full ESC/ERS guidelines provided only a modest benefit to TRV alone [5].

Almost a third of TTEs did not have sufficient information to determine PH probability using the ESC/ERS guidelines, suggesting that the recommended parameters are either difficult to obtain, even in PH centres, or not widely used, or a combination of both. Category B signs (pulmonary artery) were most often missing from TTE reports, consistent with a previous study [8]. A simplification of the ESC/ERS algorithm may be warranted. While we cannot make definitive recommendations, our data suggest that RV:LV ratio, mid‐systolic notching and right atrial area would be good candidates as they were identified as significant predictors of PH in a logistic regression analysis. Other support for inclusion of these parameters include that RV:LV ratio and right atrial area were recorded on the majority of TTEs and that D'Alto et al. also found RV:LV ratio to be predictive of PH at univariate analysis [5].

### 4.3 Performance of NT‐proBNP

Studies on the diagnostic utility of NT‐proBNP in PH have suggested different cut‐offs [9, 10, 11, 12, 21, 22, 23, 24, 25, 26]. Therefore, we defined optimal cut‐points from a ROC analysis of our data. The AUC (0.84) and the performance of the derived NT‐proBNP cut‐points (sensitivity 75%–83% and specificity 69%–86%) were reasonably close to those of the echocardiographic measures. Notably, the F1 cut‐point (maximizing sensitivity and PPV) of NT‐proBNP > 138.9 ng/L only differed to the full ESC/ERS guidelines in its classification of around 20 patients. We also investigated the ability of NT‐proBNP to detect PH in 167 patients who were non‐assessable or had low PH probability by the ESC/ERS algorithm (and had an NT‐proBNP measurement); NT‐proBNP > 80.2 ng/L identified 52 PH patients who may otherwise have been missed but failed to flag 17 PH patients. These results highlight the importance of testing NT‐proBNP levels, especially in cases where TRV cannot be measured, and that NT‐proBNP levels that fall within the normal range may still warrant additional testing in some cases.

NT‐proBNP is included in the validated DETECT risk score to screen for PAH in systemic sclerosis (SSc) [27] and other studies suggest it has value as a diagnostic biomarker in other groups at high risk of PH [21] (although many of these studies use TTE as well as – or instead of – RHC to ‘diagnose’ PH) [11, 21, 23, 24]. Our study includes all WHO PH groups and suggests NT‐proBNP has a more important role in detecting PH than currently appreciated. It may be useful as an initial test in all breathless patients without left heart disease for whom there is a lower suspicion of PH, or it could be used in resource‐constrained settings to identify patients who urgently need further work‐up. Importantly, NT‐proBNP could not be used to rule out PH as it is a biomarker of heart failure and levels can therefore be low in early‐stage or well‐controlled PH [1].

### 4.4 Unmet Need for Additional Investigative Tools

Even with most TTEs conducted in expert centres, 42% of patients with low or non‐assessable probability of PH (by the ESC/ERS guidelines) did have PH, thus demonstrating the need for additional investigative tools, especially for patients with inconspicuous echocardiographic signs of PH. While these patients may have had other PH risks leading to the relatively high prevalence of PH with low or non‐assessable risk echocardiograms, Slegg et al. found a similarly high prevalence, with 36% of those with a low probability echocardiogram having PH [8].

Moreover, in other studies without a specialist conducting or reading TTEs, PH is often missed even when echocardiographic signs are apparent [28, 29]. One avenue to address this unmet need may be to combine TTE parameters with non‐echocardiographic measures to improve sensitivity. Other groups are refining the predictive ability of readily accessible tests with artificial intelligence to improve detection [4, 30, 31].

### 4.5 Strengths and Limitations

This multicentre, international, prospective study has several important strengths, including that all 475 newly referred patients received TTE and RHC, regardless of their TTE result, in contrast to previous studies [5, 7, 8], almost all TTEs were performed around the time of RHC and all were centrally read in a blinded manner. Limitations include that CIPHER was conducted prior to the release of the 2022 ESC/ERS guidelines [1] and, consequently, tricuspid annular plane systolic excursion/systolic pulmonary artery pressure (TAPSE/sPAP) ratios were not collected and we cannot fully assess the performance of the latest ESC/ERS TTE algorithm. The prevalence of PH (73% mPAP > 20 mmHg) is much higher than that of patients presenting with chronic unexplained dyspnoea in the general population (1% of general population with mPAP ≥ 25 mmHg) [32]. The high levels of missing data mean we cannot assess the full 2015 ESC/ERS TTE algorithm performed exactly as instructed. However, the missing data reflect real‐world clinical practice and therefore support the ultimate aim of the study. Future work to assess the performance of TTE for detection of PH would benefit from capturing the reasons for missing data (e.g., early disease, inadequate tricuspid regurgitation, difficulties in obtaining TTE due to obesity or other factors, non‐specialist operator) to understand how the use of TTE can be improved.

### 4.6 Conclusion

The findings from this prospective study of 475 patients who were newly referred to PH centres and all underwent RHC demonstrate the limitations of echocardiographic investigation of PH. The performance of the full 2015 ESC/ERS TTE algorithm was similar to that of RVSP > 40 mmHg (sensitivity and specificity 77%–79%). Among patients who were classed as low probability (22 patients by ESC/ERS algorithm and 110 by RVSP) or non‐assessable due to missing data (151 patients by ESC/ERS and 73 by RVSP), over 40% had PH. Additional testing for whether patients' NT‐proBNP measurements were > 80.2 ng/L would identify a further 52 true PH cases. NT‐proBNP alone, however, cannot reliably exclude PH. Other, more effective, non‐invasive PH investigative tools are needed.

## Author Contributions

**Luke S. Howard:** conceptualisation, investigation, methodology, writing – review and editing. **David G. Kiely:** conceptualisation, investigation, methodology, writing – review and editing. **Allan Lawrie:** conceptualisation, investigation, methodology, writing – review and editing. **Bradley A. Maron:** conceptualisation, investigation, methodology, writing – review and editing. **Ioana R. Preston:** conceptualisation, investigation, methodology, writing – review and editing. **Stephan Rosenkranz:** conceptualisation, investigation, methodology, writing – review and editing. **Mark Toshner:** conceptualisation, investigation, methodology, writing – review and editing. **Martin R. Wilkins:** conceptualisation, investigation, methodology, writing – review and editing. **Yiu‐Lian Fong:** conceptualisation, investigation, methodology, writing – review and editing. **Debbie Quinn:** conceptualisation, investigation, methodology, project administration, supervision, writing – review and editing. **Dimitri Stamatiadis:** conceptualisation, investigation, methodology, project administration, supervision, writing – review and editing. **Matthieu Villeneuve:** conceptualisation, data curation, formal analysis, investigation, methodology, writing – review and editing. **Kelly M. Chin:** conceptualisation, investigation, methodology, writing – review and editing.

## Ethics Statement

The protocol and other study documentation were reviewed and approved by the relevant Institutional Review Board/Independent Ethics Committee for each site before the study was initiated (Supplemental Table 3) and each patient gave written informed consent.

## Conflicts of Interest

Luke Howard has served as a member of the CIPHER steering committee for Johnson & Johnson, Gossamer Bio and Lung Biotechnology; has received consulting fees from Altavant; has received research grants from Johnson & Johnson; has received speaker fees from Bayer PLC, Johnson & Johnson and Merck; has received support for attending meetings and/or travel from Johnson & Johnson; has been a member of an advisory board for Acceleron, Johnson & Johnson and Merck; and is a shareholder in iOWNA, Circular, ATXA Therapeutics and Calibre Biometrics. David G. Kiely has served as a member of the CIPHER steering committee for Johnson & Johnson and his institution has received support from National Institute of Health Research Sheffield Biomedical Research Centre as part of the support for the present manuscript. He has received consulting fees and honoraria for lectures, presentations, speakers bureaus, manuscript writing or educational events from Johnson & Johnson, Ferrer, Altavant, MSD and United Therapeutics; has received support for attending meetings and/or travel from Johnson & Johnson, Ferrer, MSD and United Therapeutics; has participated on a Data Safety Monitoring Board of Advisory Board for Johnson & Johnson and MSD; is a member of the Clinical Reference Group for Specialised Respiratory Medicine (NHS England, unpaid); and is the lead of the UK National Audit of Pulmonary Hypertension (paid). David G. Kiely's institution has received grants or contracts from Johnson & Johnson, National Institute of Health Research Sheffield Biomedical Research Centre and Ferrer. Allan Lawrie has received payment for serving as a steering committee member for Johnson & Johnson (as part of the support for the present manuscript) and has received support for attending meetings and/or travel from Johnson & Johnson. He receives funding from the British Heart Foundation through a Senior Basic Science Research Fellowship (FS/18/52/33808), the Imperial British Heart Foundation Imperial Centre for Research Excellence (RE/18/4/34215), Alexion Pharmaceuticals and Apple Inc (Investigator Awards). Bradley A. Maron has served as a member of the CIPHER steering committee for Johnson & Johnson; and has received consultancy fees from Actelion Pharmaceuticals, Tenax and Regeneron and grants from Deerfield, Boston Biomedical Innovation Center and the Cardiovascular Medical Research Education Foundation. He has two patents pending and one patent issued relevant to the submitted work. He has served as PI or co‐PI on various projects: 5R01HL139613‐03: PI on NIH R01 award focusing on molecular mechanisms that regulate vascular fibrosis in pulmonary arterial hypertension ($1,748,134); NIH R01HL163960: Co‐PI on NIH R01 award using network medicine to prognosticate patients with pulmonary hypertension ($286,861); U54HL119145 and Boston Biomedical Innovation Center (BBIC): PI on NIH‐funded project to develop an antibody therapeutic for chronic thromboembolic pulmonary hypertension ($341,589); Brigham IGNITE award: PI on project to develop an antibody therapeutic for chronic thromboembolic pulmonary hypertension ($50,000); NIH R01HL153502: PI on NIH‐funded project to clarify the mechanisms regulating NEDD9‐SMAD3 interactions in thrombotic vascular disease ($864,664); NIH R01HL155096‐01: PI on NIH‐funded project to clarify individualize the pathophenotype of patients with pulmonary hypertension ($809,353). Ioana R. Preston has served as a member of the CIPHER steering committee for Johnson & Johnson, Merck, Liquidia; she has received consulting fees and honoraria for lectures, presentations, manuscript writing or educational events from Johnson & Johnson, Altavant, Gossamer and United Therapeutics; has received support for attending meetings and/or travel from Johnson & Johnson, Merck and United Therapeutics; Ioana Preston's institution has received grants or contracts from Johnson & Johnson, Merck, United Therapeutics, Respira and Bellerophon. Stephan Rosenkranz has served as a steering committee member for Johnson & Johnson (as part of the support for the present manuscript); has received remunerations for lectures and/or consultancy from Abbott, Acceleron, Actelion, Aerovate, Altavant, AOP, AstraZeneca, Bayer, Boehringer‐Ingelheim, Edwards, Ferrer, Gossamer, Janssen, Lilly, MSD, United Therapeutics, Vifor. His institution has received research grants from Actelion, AstraZeneca, Bayer, Johnson & Johnson and Lempo. Mark Toshner has served as a steering committee member for Johnson & Johnson (as part of the support for the present manuscript), has received support for attending meetings and/or travel from Johnson & Johnson & GSK and has been a member of an advisory board for MorphogenIX. Martin R Wilkins has served as a member of the CIPHER steering committee for Johnson & Johnson and his institution received clinical research facility and Biomedical Research Centre infrastructure support from National Institute of Health Research Sheffield Biomedical Research Centre as part of the support for the present manuscript. He has received consulting fees from MorphogenIX, VIVUS, Johnson & Johnson, Kindaset, Chiesi, Aerami and BenevolentAI and has patents planned, issued and/or pending with Imperial Innovations (patent submitted for prognostic protein model and diagnostic miRNA model and patent for ZIP12 as a drug target); has participated in an adjudication committee for three clinical trials for Acceleron and in a study safety committee for GSK. Martin R. Wilkins' institute has received grants or contracts from British Heart Foundation (RE/18/4/34215 centre support). Yiu‐Lian Fong was an employee of Johnson & Johnson at the time of study and owns shares of stock/stock options in Johnson & Johnson. Debbie Quinn, Dimitri Stamatiadis and Matthieu Villeneuve are employees of Johnson & Johnson, and own shares of stock/stock options in Johnson & Johnson. Kelly M Chin has received payment for work on steering, advisory or adjudication committee work with Arena, Bayer, Gossamer Bio, Johnson & Johnson and Merck, payment for consulting work with Altavant, and her institution has received research support for clinical studies overseen by her from Altavant, Gossamer Bio, Johnson & Johnson, Merck, Pfizer and United Therapeutics.

## Supporting information


**Supporting Figure 1:** Examples of hypothetical TTEs from patients with intermediate probability of PH (A), low probability of PH (B) and non‐assessable probability of PH (C) as defined using the 2015 ESC/ERS TTE algorithm. **Supporting Table 1:** Eligibility criteria. Supplemental Table 2. WHO PH Group definitions used to classify patients in CIPHER study. Supplemental Table 3. IRB and EC approvals. Supplemental Table 4. Reasons for having a TTE image with poor resolution. Supplemental Table 5. Results of logistic regression model and Wald chi‐squared test to examine the contribution of the seven non‐TRV echocardiographic parameters in predicting PH, when combining missing values with normal values. Supplemental Table 6. Results of logistic regression model and Wald chi‐squared test to examine the contribution of the seven non‐TRV echocardiographic parameters in predicting PH, when combining missing values with abnormal values. Supplemental Table 7. Baseline characteristics of PH and non‐PH patients, by PH definition. Supplemental Table 8. Performance of echocardiographic parameters in distinguishing PH (mPAP ≥ 25 mmHg) from non‐PH (mPAP < 25 mmHg). Supplemental Table 9. Baseline characteristics of pre‐capillary and non‐pre‐capillary PH patients. Supplemental Table 10. Performance of echocardiographic parameters in distinguishing pre‐capillary PH (mPAP > 20 mmHg and PVR ≥ 3 WU) from non‐pre‐capillary PH (mPAP ≤ 20 mmHg and/or PVR < 3WU). **Supplemental Appendix 1:** Classification of patients by the 2015 ESC/ERS TTE algorithm. **Supplemental Appendix 2:** Evaluation of the contribution of each of the echocardiographic parameters recommended by the 2015 European Society of Cardiology (ESC)/European Respiratory Society (ERS) guidelines for determining probability of pulmonary hypertension (PH). **Supplemental Appendix 3:** Statistical analysis.

## Data Availability

Although these data are not currently publicly available for sharing, requests can be sent to the corresponding author and will be evaluated on an individual basis.
