# Supplementary material for: Performance of Guideline‐Recommended Approaches to Echocardiographic Investigation for Pulmonary Hypertension: Analysis of the CIPHER Study
Source: Pulm Circ. 2026 Feb 17;16(1):e70258. doi: 10.1002/pul2.70258 (PMC12910320; doi:10.1002/pul2.70258)
Supplement: Supplementary file 1 — Supporting Figure 1: Examples of hypothetical TTEs from patients with intermediate probability of PH (A), low probability of PH (B) and non‐assessable probability of PH (C) as defined using the 2015 ESC/ERS TTE algorithm. Supporting Table 1: Eligibility criteria. Supplemental Table 2. WHO PH Group definitions used to classify patients in CIPHER study. Supplemental Table 3. IRB and EC approvals. Supplemental Table 4. Reasons for having a TTE image with poor resolution. Supplemental Table 5. Results of logistic regression model and Wald chi‐squared test to examine the contribution of the seven non‐TRV echocardiographic parameters in predicting PH, when combining missing values with normal values. Supplemental Table 6. Results of logistic regression model and Wald chi‐squared test to examine the contribution of the seven non‐TRV echocardiographic parameters in predicting PH, when combining missing values with abnormal values. Supplemental Table 7. Baseline characteristics of PH and non‐PH patients, by PH definition. Supplemental Table 8. Performance of echocardiographic parameters in distinguishing PH (mPAP ≥ 25 mmHg) from non‐PH (mPAP < 25 mmHg). Supplemental Table 9. Baseline characteristics of pre‐capillary and non‐pre‐capillary PH patients. Supplemental Table 10. Performance of echocardiographic parameters in distinguishing pre‐capillary PH (mPAP > 20 mmHg and PVR ≥ 3 WU) from non‐pre‐capillary PH (mPAP ≤ 20 mmHg and/or PVR < 3WU). Supplemental Appendix 1: Classification of patients by the 2015 ESC/ERS TTE algorithm. Supplemental Appendix 2: Evaluation of the contribution of each of the echocardiographic parameters recommended by the 2015 European Society of Cardiology (ESC)/European Respiratory Society (ERS) guidelines for determining probability of pulmonary hypertension (PH). Supplemental Appendix 3: Statistical analysis. [file PUL2-16-e70258-s001.docx]

**SUPPLEMENTAL MATERIAL**

**Performance of guideline-recommended approaches to echocardiographic investigation for pulmonary hypertension: analysis of the CIPHER study**

Prof. Luke S. Howard^1^, Prof. David G. Kiely^2^, Prof. Allan Lawrie^3^, Prof. Bradley A. Maron^4,5^, Associate Prof. Ioana R. Preston^6^, Prof. Stephan Rosenkranz^7^, Dr Mark Toshner^8^, Prof. Martin R Wilkins^3^, Dr Yiu-Lian Fong^9^, Dr Debbie Quinn^10^, Dr Dimitri Stamatiadis^10^, Mr Matthieu Villeneuve^10^, Prof. Kelly M Chin^11^

^1^National Pulmonary Hypertension Service, Hammersmith Hospital, London, UK.

^2^Sheffield Pulmonary Vascular Disease Unit and NIHR Biomedical Research Centre, Royal Hallamshire Hospital, Sheffield Teaching Hospitals NHS Foundation Trust, Sheffield, UK.

^3^National Heart and Lung Institute, Imperial College London, London, UK.

^4^Department of Medicine, University of Maryland School of Medicine, Baltimore, MD, USA.

^5^University of Maryland-Institute for Health Computing, Bethesda, MD, USA.

^6^ Department of Pulmonary and Critical Care Medicine, Lahey Hospital and Medical Center.^7^Department of Cardiology and Cologne Cardiovascular Research Center (CCRC), Heart Center, University Hospital Cologne, Cologne, Germany.

^8^Department of Medicine, University of Cambridge, Cambridge, UK.

^9^Johnson & Johnson, NJ, USA.

^10^Johnson & Johnson, Allschwil, Switzerland.

^11^Division of Pulmonary and Critical Care Medicine, UT Southwestern Medical Center, Dallas, TX, USA.

**Address for correspondence:**

Prof. Luke Howard, MD

National Pulmonary Hypertension Service,

Hammersmith Hospital, 72 Du Cane Rd,

W12 0HS, London, UK.

Tel: +44 20 3313 3171; Email: [l.howard@imperial.ac.uk](mailto:l.howard@imperial.ac.uk)

**Supplemental Table 1**. Eligibility criteria.

| **Inclusion criteria** |
| --- |
| - Male or female - Aged ≥18 years of age inclusive - Have undergone or planned RHC within 6 weeks - Medically stable on the basis of physical examination, medical history and vital signs performed at screening. Any abnormalities must be consistent with the underlying illness in the study population and this determination must be recorded in the participant’s source documents and initiated by the investigator - Must sign an ICF (or their legally acceptable representative must sign) indicating that he or she understands the purpose of, and procedures required for, the study and is willing to participate in the study |
| **Exclusion criteria**   - Participants requiring renal dialysis - History of heart or lung transplant (waiting list status or consideration of enlisting is allowed) - Severe left ventricular dysfunction (left ventricular ejection fraction <35%) - Ongoing contagious respiratory disease - Participants that have previously contributed blood samples for biomarker analysis in the Actelion retrospective study of biomarkers for PH - Participants unable to have at least 2 tubes of 10 mL blood drawn - Treatment with any PAH-specific drug prior to collection of biomarker samples |
|  |
|  |
|  |
|  |
|  |
|  |
|  |

ICF, informed consent form; PAH, pulmonary arterial hypertension; PH, pulmonary hypertension; RHC, right heart catheterization.

**Supplemental Table 2**. WHO PH Group definitions used to classify patients in CIPHER study.

| **The number of participants to be enrolled will be limited based on the following group definitions:** |
| --- |
| **Non-PH (194 participants)** |
| - Participants who underwent RHC for suspicion of PH due to atypical shortness of breath, or suspicion of PH associated with left ventricular dysfunction, systolic or diastolic, valvular heart disease, lung disease or pulmonary embolism but were found to have rest mPAP <21 mmHg |
| **PAH (Group 1 PH, 194 participants)** |
| - Participants with   - Rest mPAP >20 mmHg, PCWP ≤15 mmHg; PVR ≥3 Wood Units   - FVC ≥70% and FEV1 ≥60%   - Absence or only modest airway or parenchymal abnormalities   - No evidence of CTEPH or diseases associated with group 5 PH   - Belong to one of the sub-groups:     - Idiopathic PAH     - Heritable PAH     - Drug- and toxin-induced PAH     - PAH associated with connective tissue disease, HIV, portal hypertension, congenital heart disease or schistosomiasis     - PAH long-term responder to calcium channel blockers     - PAH with overt features of venous/capillaries (pulmonary veno-occlusive disease/pulmonary capillary hemangiomatosis) involvement |
| **CTEPH (Group 4 PH, 194 participants)** |
| - Participants with:   - Rest mPAP > 20 mmHg, PCWP ≤15 mmHg, PVR ≥3 Wood Units   - VQ scan, CT angiogram or angiogram consistent with chronic thromboembolism, and   - More than 3 months of anticoagulation therapy at any time   - No PEA or BPA post RHC (may have had PEA or BPA prior to RHC) |
| **Group 2, 3 or 5 PH (194 participants)** |
| - **Group 2 PH** participants with:   - IpcPH with rest mPAP > 20 mmHg, PCWP >15 mmHg and PVR <3 Wood Units or   - CpcPH with rest mPAP >20 mmHg, PCWP >15 mmHg and PVR ≥3 Wood Units   - Left ventricular dysfunction (systolic, diastolic or valvular heart disease, HFrEF, HFpEF, VHD, RCM, HCM). - **Group 3 PH** participants with:   - Rest mPAP >20 mmHg, PCWP ≤15 mmHg, PVR ≥3 Wood Units   - Moderate restrictive and/or obstructive lung disease with FVC <70% and/or FEV1 <60% and characteristic airway and/or parenchymal abnormalities, or   - Obstructive sleep apnoea as documented by polysomnography - **Group 5 PH** participants with:   - Rest mPAP > 20 mmHg, PCWP ≤ 15 mmHg, PVR ≥ 3 Wood Units or IpcPH or CpcPH (as defined above under Group 2 PH) and with associated diseases including, but not limited to:     - Hematologic disorders     - Complex congenital heart diseases     - Systemic and metabolic disorders |

BPA, balloon pulmonary angiogram; CpcPH, combined pre- and post-capillary PH; CT, computed tomography; CTEPH, chronic thromboembolic pulmonary hypertension; FEV1, forced expiratory volume in 1 second; FVC, forced vital capacity; HCM, hypertrophic cardiomyopathy; HFrEF, heart failure with reduced ejection fraction; HFpEF, heart failure with preserved ejection fraction; HIV, human immunodeficiency virus; IpcPH, isolated post-capillary PH; mPAP, mean pulmonary artery pressure; PAH, pulmonary arterial hypertension; PEA, pulmonary endarterectomy; PCWP, pulmonary capillary wedge pressure; PH, pulmonary hypertension; PVR, pulmonary vascular resistance; RCM, restrictive cardiomyopathy; RHC, right heart catheterization; VHD, valvular heart disease; VQ, ventilation perfusion; WHO, World Health Organization.

**Supplemental Table 3.** IRB and EC approvals.

| **Country of site** | **IRB/EC** |
| --- | --- |
| Belgium  France  Germany  Germany  Germany  Germany  Germany  Germany  Germany  Netherlands  Poland  Poland  Poland  Spain  Ukraine  Ukraine  Ukraine  Ukraine  USA  USA  USA  USA  USA  USA  USA USA USA USA USA USA USA | Commissie Medische Ethiek UZ Gasthuisberg |
|  | CPP Ouest III |
|  | Ethik-Kommission an der TU Dresden  Ethikkommission an der Medizinischen Fakultaet der Rheinischen Friedrich-Wilhelms-Universitaet Bonn  Ethikkommission d. Friedrich-Schiller Universität  Ethikkommission der Medizinischen Fakultät der Universität Heidelberg  Ethik-Komission der Universität zu Lübeck  Ethikkommission des Fachbereichs Medizin der Justus-Liebig-Universitat Giessen  Ethikkommission bei der Landesärztekammer Hessen  METc VUmc  Komisja Bioetyczna Pomorskiego Uniwersytetu Medycznego w Szczecinie  Komisja Bioetyczna Centrum Medyczne Kształcenia Podyplomowego  Komisja Bioetyczna przy Lubelskiej Izbie Lekarskiej  CEIm Regional de la Comunidad de Madrid  Ethics Commission of SI''National Scientific Center ''The M.Strazhesko Institute of Cardiology of NAMSU''  Ethics Committee of Communal Noncommercial Enterprise "Cherkasy Regional Cardiological Center of Cherkasy Regional Council"  Local Ethics Committee of Public Institution "F.H. Yanovskyi Phthisiology and Pulmonology National Institute under the Ukrainian Academy of Medical Sciences"  Ethics Committee of Communal Enterprise "Dnipropetrovsk Regional Clinical Centre of Cardiology and Cardiosurgery" of Dnipropetrovsk Regional Council  University of Texas Southwestern Medical Center IRB/EC  University of Cincinnati Institutional Review Board  Sterling IRB  Colorado Multiple Institutional Review Board  Sterling IRB  University of Southern California IRB  Houston Methodist Hospital IRB  University of Iowa IRB-1 Biomedical  St. Vincent's Institutional Review Board  WIRB  Mayo Clinic IRB  IRB Office of Regulatory Affairs  Louisiana State University Health Sciences Center (New Orleans, LA, USA) |

EC, ethics committee; IRB, institutional review board.

**Supplemental Table 4.** Reasons for having a TTE image with poor resolution.

| **Reason** | **N** |
| --- | --- |
| Total number of TTEs with poor quality assessment | 379 |
| Incomplete anatomical coverage  Other  Poor resolution  Improper gain  Insufficient cardiac cycles  Motion artefacts  Incorrect formatting  ECG signal missing  No reason given  Duplicate submission  Inappropriate focus zone  Acoustic artefacts  Improper frame rate/sweep speed  Inappropriate probe  ECG signal obscuring image  Missing image scale | 298 |
|  | 193 |
|  | 178  99  85  67  19  16  13  10  6  2  2  2  1  1 |

ECG, electrocardiogram; TTE, transthoracic echocardiogram.

**Supplemental Table 5.** Results of logistic regression model and Wald chi-squared test to examine the contribution of the seven non-TRV echocardiographic parameters in predicting PH, when combining missing values with normal values.

| **Parameter** | **Analysis of maximum likelihood estimates** | | | | | |
| --- | --- | --- | --- | --- | --- | --- |
|  |  | **DF** | **Estimate** | **Standard error** | **Wald  chi-squared** | **Two-sided  *P* value** |
| **Intercept** |  | 1 | 8.7098 | 348.3 | 0.0006 | 0.9801 |
| **Right ventricle/left ventricle basal diameter ratio <1.0** | N | 1 | −0.9929 | 0.2803 | 12.5485 | 0.0004 |
| **Left ventricular eccentricity index >1.1 in systole and/or diastole** | N | 1 | −0.8834 | 0.5317 | 2.7607 | 0.0966 |
| **Right ventricular outflow  Doppler acceleration time <105 msec** | N | 1 | −0.0634 | 0.1603 | 0.1566 | 0.6923 |
| **Mid-systolic notching** | N | 1 | −0.9782 | 0.3359 | 8.4818 | 0.0036 |
| **Early diastolic pulmonary regurgitation velocity >2.2 m/sec** | N | 1 | −4.9368 | 348.3 | 0.0002 | 0.9887 |
| **Pulmonary artery diameter >25 mm** | N | 1 | 0.0649 | 0.1619 | 0.1607 | 0.6885 |
| **Right atrial area (end-systole) >18 cm^2^** | N | 1 | −0.4344 | 0.1435 | 9.1671 | 0.0025 |
| **Inferior cava diameter >21 mm with decreased inspiratory collapse (<50% with a sniff or <20% with quiet inspiration)** | N | 1 | −0.5455 | 0.5474 | 0.9931 | 0.3190 |

The parameters were included in the model after categorization, as there were too many missing values to run regression models using variables in their continuous form. This table shows results when missing values are combined with normal values. DF, degrees of freedom; N, no abnormality; PH, pulmonary hypertension; TRV, tricuspid regurgitation velocity.

**Supplemental Table 6.** Results of logistic regression model and Wald chi-squared test to examine the contribution of the seven non-TRV echocardiographic parameters in predicting PH, when combining missing values with abnormal values.

| **Parameter** | **Analysis of maximum likelihood estimates** | | | | | |
| --- | --- | --- | --- | --- | --- | --- |
|  |  | **DF** | **Estimate** | **Standard error** | **Wald  chi-squared** | **Two-sided  *P* value** |
| **Intercept** |  | 1 | 1.2058 | 0.2385 | 25.5555 | <0.0001 |
| **Right ventricle/left ventricle basal diameter ratio <1.0** | N | 1 | −0.4374 | 0.1340 | 10.6526 | 0.0011 |
| **Left ventricular eccentricity index >1.1 in systole and/or diastole** | N | 1 | 0.1550 | 0.1274 | 1.4814 | 0.2236 |
| **Right ventricular outflow  Doppler acceleration time <105 msec** | N | 1 | −0.1899 | 0.1914 | 0.9843 | 0.3211 |
| **Mid-systolic notching** | N | 1 | −0.3543 | 0.1547 | 5.2421 | 0.0220 |
| **Early diastolic pulmonary regurgitation velocity >2.2 m/sec** | N | 1 | 0.6706 | 0.1880 | 12.7261 | 0.0004 |
| **Pulmonary artery diameter >25 mm** | N | 1 | 0.0479 | 0.1495 | 0.1029 | 0.7484 |
| **Right atrial area (end-systole) >18 cm^2^** | N | 1 | −0.2706 | 0.1359 | 3.9655 | 0.0464 |
| **Inferior cava diameter >21 mm with decreased inspiratory collapse (<50% with a sniff or <20% with quiet inspiration)** | N | 1 | 0.1584 | 0.1413 | 1.2575 | 0.2621 |

The parameters were included in the model after categorization, as there were too many missing values to run regression models using variables in their continuous form. This table shows results when missing values are combined with abnormal values. DF, degrees of freedom; N, no abnormality; PH, pulmonary hypertension; TRV, tricuspid regurgitation velocity.

**Supplemental Table 7.** Baseline characteristics of PH and non-PH patients, by PH definition.

| **Characteristic** | **PH defined as mPAP ≥25 mmHg** | | **PH defined as mPAP >20 mmHg** | |
| --- | --- | --- | --- | --- |
|  | **PH**  **(n=296)** | **Non-PH**  **(n=179)** | **PH (n=345)** | **Non-PH (n=130)** |
| **Age, years** | 63.9 ± 13.9 | 58.2 ± 14.5 | 63.8 ± 13.8 | 56.1 ± 14.5 |
| **Female, n (%)** | 56.4 | 59.8 | 56.5 | 60.8 |
| **mPAP, mmHg** | 41.7 ± 11.2 | 17.5 ± 3.8 | 38.9 ± 12.4 | 15.7 ± 2.8 |
| **PAWP, mmHg** | 12.0 ± 5.8 | 9.2 ± 3.8 | 11.9 ± 5.6 | 8.2 ± 3.3 |
| **mRAP by RHC, mmHg** | 9.3 ± 5.2 (n=295) | 5.2 ± 3.6 | 9.0 ± 5.2 (n=344) | 4.4 ± 2.8 |
| **mRAP by TTE, mmHg**  Based on expiration diameter  Based on inspiration diameter | 13.1 ± 5.1  9.9 ± 2.5 | 11.7 ± 3.9  9.2 ± 2.0 | 13.0 ± 5.1  9.8 ± 2.5 | 11.3 ± 3.4  9.2 ± 1.9 |
| **RVSP, mmHg**  Based on expiration diameter  Based on inspiration diameter | 63.9 ± 20.9 (n=274)  60.5 ± 20.1 (n=274) | 38.1 ± 10.7 (n=128)  35.1 ± 9.9 (n=128) | 61.2 ±21.3 (n=312)  57.7 ± 20.6 (n=312) | 36.8 ± 10.4 (n=90)  34.1 ± 9.6 (n=90) |
| **NT-proBNP, ng/L** | 1866.3 ± 2162.5 | 410.4 ± 1247.0 | 1730.4 ± 2188.2 | 233.5 ± 531.6 |
| **PH group, n** | 296 | – | 345 | – |
| Group 1 | 112 (37.8%) | – | 117 (33.9%) | – |
| Group 2 | 54 (18.2%) | – | 60 (17.4%) | – |
| Group 3 | 36 (12.2%) | – | 40 (11.6%) | – |
| Group 4 | 63 (21.3%) | – | 65 (18.8%) | – |
| Group 5 | 12 (4.1%) | – | 13 (3.8%) | – |
| Unclear | 9 (3.0%) | – | 11 (3.2%) | – |
| Unclassifiable | 10 (3.4%) | – | 39 (11.3%) | – |
| **BMI, kg/m^2^** | 29.5 ± 7.3 | 28.9 ± 7.7 | 29.5 ± 7.2 | 28.8 ± 8.2 |
| **Race, n (%)** | 293 | 176 | 342 | 127 |
| Black or African American Native | 7 (2.4%) | 5 (2.8%) | 7 (2.1%) | 5 (3.9%) |
| American Indian/ Alaskan | 2 (0.7%) | 1 (0.6%) | 3 (0.9%) | 0 |
| White | 270 (92.2%) | 161 (91.5%) | 317 (92.7%) | 114 (89.8%) |
| Other | 14 (4.8%) | 9 (5.1%) | 15 (4.4%) | 8 (6.3%) |
| **New York Heart Association functional class, n (%)** | 290 | 67 | 331 | 26 |
| I | 5 (1.7%) | 5 (7.5%) | 6 (1.8%) | 4 (15.4%) |
| II | 62 (21.4%) | 24 (35.8%) | 79 (23.9%) | 7 (26.9%) |
| III | 199 (68.6%) | 37 (55.2%) | 221 (66.8%) | 15 (57.7%) |
| IV | 24 (8.3%) | 1 (1.5%) | 25 (7.6%) | 0 |

Values represent mean ± SD for continuous variables and n (%) for categorical variables.

BMI, body mass index; mPAP, mean pulmonary artery pressure; mRAP, mean right atrial pressure; NT-proBNP, N-terminal pro-brain natriuretic peptide; PAWP, pulmonary artery wedge pressure; PH, pulmonary hypertension; RHC, right heart catheterization; RVSP, right ventricle systolic pressure; SD, standard deviation; TTE, transthoracic echocardiogram; WHO FC, World Health Organization functional class.

**Supplemental Table 8.** Performance of echocardiographic parameters in distinguishing PH (mPAP ≥25 mmHg) from non-PH (mPAP <25 mmHg).

| **TTE classification** | **RHC diagnosis of PH** | | **Prevalence of PH by RHC** | **Sensitivity* [95% CI]** | **Specificity* [95% CI]** | **PPV [95% CI]** | **NPV  [95% CI]** |
| --- | --- | --- | --- | --- | --- | --- | --- |
|  | **Yes** | **No** |  |  |  |  |  |
| **Performance of full 2015 ESC/ERS TTE algorithm** | | | | | |  |  |
| High | 192 | 6 | 97.0% | **86.5%**  [82.1;89.9] |  | **84.8%**  [80.3;88.4] |  |
| Intermediate | 64 | 40 | 61.5% |  |  |  |  |
| Low | 3 | 19 | 13.6% |  | **74.3%**  [67.4;80.1] |  | **76.9%**  [70.1;82.5] |
| Non-assessable | 37 | 114 | 24.5% |  |  |  |  |
| **Performance of peak TRV alone** | | | | | |  |  |
| High (>3.4 m/s) | 143 | 4 | 97.3% | **76.7%**  [71.5;81.1] |  | **89.0%** |  |
| Intermediate (2.9–3.4 m/s) | 84 | 24 | 77.8% |  |  | [84.6;92.3] |  |
| Low (≤2.8 m/s) | 47 | 100 | 32.0% |  | **84.4%** |  | **68.6%** |
| TRV missing | 22 | 51 | 30.1% |  | [78.3;89.0] |  | [62.2;74.4] |
| **RVSP alone** | | | | | |  |  |
| High (>39 mmHg) | 246 | 54 | 82.0% | **87.8%** |  | **74.7%** |  |
| Intermediate (>33–39 mmHg) | 14 | 34 | 29.2% | [83.6;91.1] |  | [69.9;79.0] |  |
| Low (≤33 mmHg) | 14 | 40 | 25.9% |  | **50.8%** |  | **71.7%** |
| RVSP missing | 22 | 51 | 30.1% |  | [43.6;58.1] |  | [63.3;78.8] |

*For these calculations, patients who were non-assessable (by ESC/ERS guidelines) or had missing TRV/RVSP measurements were grouped with low probability patients as being PH-negative by TTE. CI, confidence interval; ESC, European Society of Cardiology; ERS, European Respiratory Society; mPAP, mean pulmonary artery pressure; NPV, negative predictive value; PH, pulmonary hypertension; PPV, positive predictive value; RHC, right heart catheterization; RVSP, right ventricular systolic pressure; TRV, tricuspid regurgitation velocity; TTE, transthoracic echocardiogram.

**Supplemental Table 9.** Baseline characteristics of pre-capillary and non-pre-capillary PH patients.

| **Characteristic** | **Pre-capillary PH defined as mPAP  >20 mmHg and PVR ≥3 WU  (6^th^ WSPH definition*)** | |
| --- | --- | --- |
|  | **PH**  **(n=263)** | **Non-PH**  **(n=212)** |
| **Age, years** | 63.0 ± 14.1 | 60.1 ± 14.6 |
| **Female, n (%)** | 56.7 | 59.0 |
| **mPAP, mmHg** | 42.3 ± 11.7 | 20.4 ± 7.8 |
| **PAWP, mmHg** | 10.5 ± 4.6 | 11.4 ± 6.0 |
| **mRAP by RHC, mmHg** |  |  |
| **NT-proBNP, ng/L** | 1923.7 ± 2212.4 | 564.4 ± 1363.7 |
| **PH group, n** | 263 | - |
| Group 1 | 117 (44.5%) | - |
| Group 2 | 26 (9.9%) | - |
| Group 3 | 40 (15.2%) | - |
| Group 4 | 61 (23.2%) | - |
| Group 5 | 10 (3.8%) | - |
| Unclear | 9 (3.4%) | - |
| Unclassifiable | 0 | - |
| **BMI, kg/m^2^** | 29.1 ± 7.0 | 29.6 ± 8.0 |
| **Race, n (%)** | 262 | 207 |
| American Indian/Alaskan | 3 (1.2%) | 0 |
| Asian | 4 (1.6%) | 2 (0.9%) |
| Black or African American Native | 6 (2.3%) | 6 (2.9%) |
| Native Hawaiian or other Pacific Islander | 1 (0.4%) | 0 |
| White | 239 (91.2%) | 192 (92.8%) |
| Not reported | 8 (3.2%) | 7 (3.2) |
| Multiple | 1 (0.4%) | 0 |
| **WHO functional class, n (%)** | 258 | 99 |
| I | 4 (1.6%) | 6 (6.1%) |
| II | 49 (19.0%) | 37 (37.4%) |
| III | 181 (70.2%) | 55 (55.6%) |
| IV | 24 (9.3%) | 1 (1.0%) |

Values represent mean ± SD for continuous variables and n (%) for categorical variables.

*Excludes isolated post-capillary PH.

BMI, body mass index; mPAP, mean pulmonary artery pressure; mRAP, mean right atrial pressure; NT-proBNP, N-terminal pro-brain natriuretic peptide; PAWP, pulmonary artery wedge pressure; PH, pulmonary hypertension; RHC, right heart catheterization; RVSP, right ventricle systolic pressure; SD, standard deviation; TTE, transthoracic echocardiogram; WHO FC, World Health Organization functional class.

**Supplemental Table 10.** Performance of echocardiographic parameters in distinguishing pre-capillary PH (mPAP >20 mmHg and PVR ≥3 WU) from non-pre-capillary PH (mPAP ≤20 mmHg and/or PVR <3WU).

| **TTE classification** | **RHC diagnosis of PH** | | **Prevalence of PH by RHC** | **Sensitivity* [95% CI]** | **Specificity* [95% CI]** |
| --- | --- | --- | --- | --- | --- |
|  | **Yes** | **No** |  |  |  |
| **Performance of full 2015 ESC/ERS TTE algorithm** | | | | | |
| High | 182 | 16 | 91.9% | 86.7%  [82.1; 90.3] |  |
| Intermediate | 46 | 58 | 44.2% |  |  |
| Low | 3 | 19 | 13.6% |  | 65.1%  [58.5; 71.2] |
| Non-assessable | 32 | 119 | 12.2% |  |  |
| **Performance of peak TRV alone** | | | | | |
| High (>3.4 m/s) | 139 | 8 | 94.6% | 79.8%  [74.6; 84.3] |  |
| Intermediate (2.9–3.4 m/s) | 71 | 37 | 65.7% |  |  |
| Low (≤2.8 m/s) | 34 | 113 | 23.1% |  | 78.8% |
| TRV missing | 19 | 54 | 26.0% |  | [72.8; 83.7] |

*For these calculations, patients who were non-assessable (by ESC/ERS guidelines) or had missing TRV/RVSP measurements were grouped with low probability patients as being PH-negative by TTE. CI, confidence interval; ESC, European Society of Cardiology; ERS, European Respiratory Society; mPAP, mean pulmonary artery pressure; NPV, negative predictive value; PH, pulmonary hypertension; PPV, positive predictive value; RHC, right heart catheterization; RVSP, right ventricular systolic pressure; TRV, tricuspid regurgitation velocity; TTE, transthoracic echocardiogram.

**Supplemental Figure 1.** Examples of hypothetical TTEs from patients with intermediate probability of PH (A), low probability of PH (B) and non-assessable probability of PH (C) as defined using the 2015 ESC/ERS TTE algorithm.


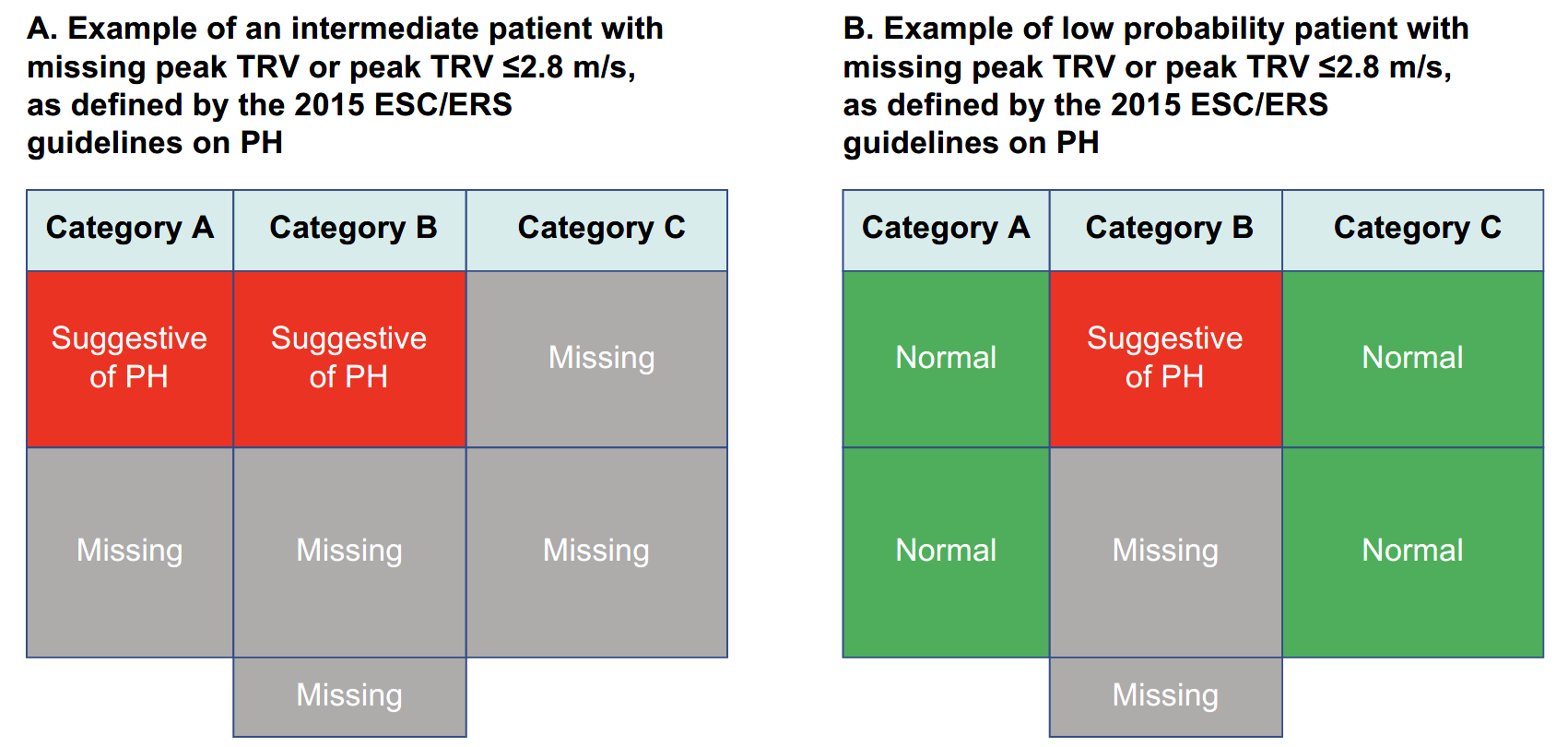


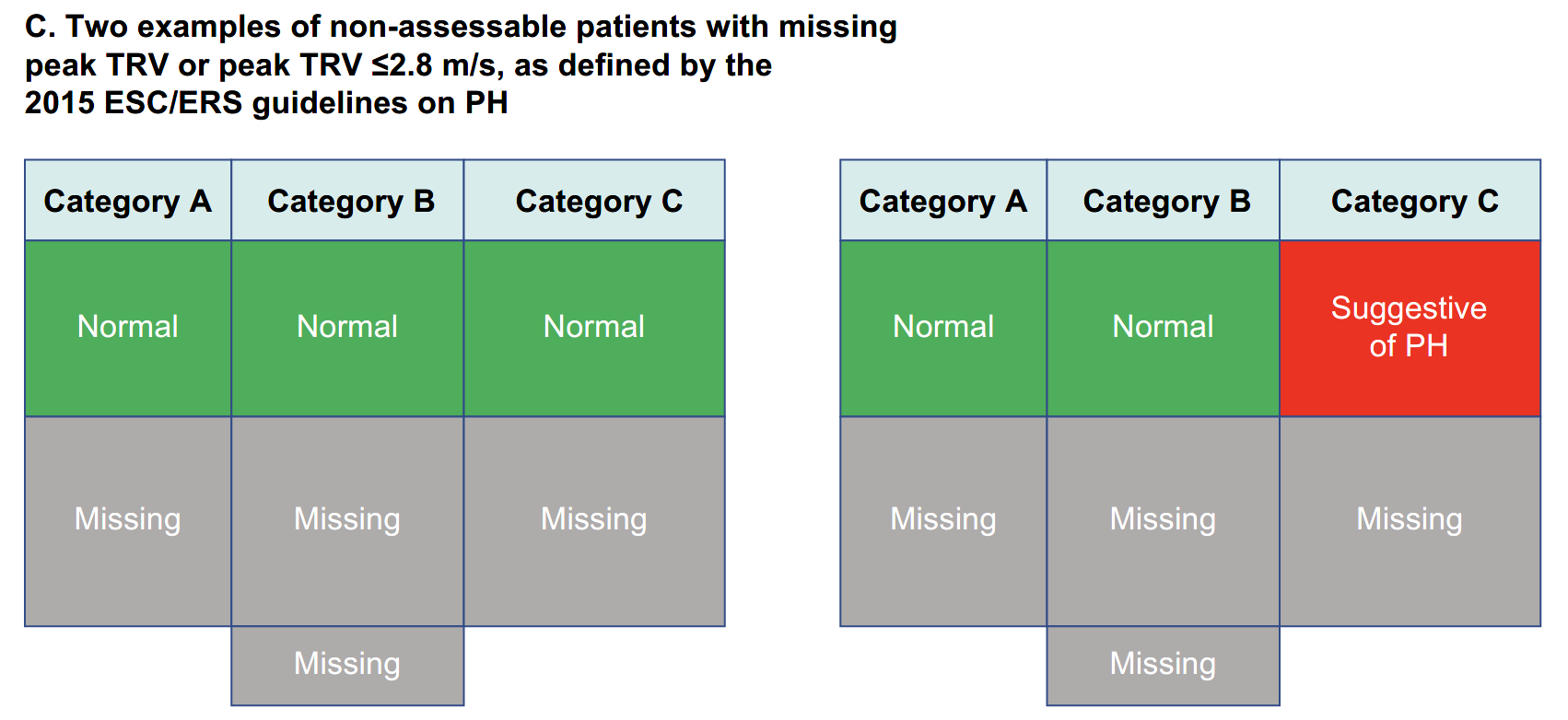


Category A signs of PH (i.e., signs in the ventricles) as right ventricle/left ventricle basal diameter ratio >1.0, flattening of the interventricular septum (left ventricular eccentricity index >1.1 in systole and/or diastole);
Category B signs of PH (i.e. signs in the pulmonary artery) as right ventricular outflow Doppler acceleration time <105 msec and/or mid-systolic notching, early diastolic pulmonary regurgitation velocity >2.2 m/sec, pulmonary artery diameter >25 mm;
Category C signs of PH (i.e., signs in the inferior vena cava and right atrium) as inferior cava diameter >21 mm with decreased inspiratory collapse (<50% with a sniff or <20% with quiet inspiration), right atrial area (end-systole) >18 cm^2^.

ERS, European Respiratory Society; ESC, European Society of Cardiology; PH, pulmonary hypertension; TRV, tricuspid regurgitation velocity; TTE, transthoracic echocardiogram.

**Supplemental Appendix 1**. Classification of patients by the 2015 ESC/ERS TTE algorithm.

To assess the performance of the 2015 European Society of Cardiology/ European Respiratory Society (ESC/ERS) transthoracic echocardiogram (TTE) algorithm, all patients were classified by their probability of pulmonary hypertension (PH) according to their peak tricuspid regurgitation velocity (TRV) measurement and whether they had signs from two or more categories of other echocardiographic signs, as per the guideline recommendations (**Figure 1A**).^1^ The total list of other (non-TRV) echocardiographic signs of PH from the 2015 ESC/ERS guidelines were as follows: category A signs were: (i) right ventricle (RV)/left ventricle (LV) basal diameter ratio >1.0 and (ii) flattening of the interventricular septum (LV eccentricity index >1.1 in systole and/or diastole); category B signs were (i) RV outflow Doppler acceleration time <105 msec and/or mid-systolic notching, (ii) early diastolic pulmonary regurgitation velocity >2.2 m/sec and (iii) pulmonary artery diameter >25 mm; category C signs were (i) inferior cava diameter >21 mm with decreased inspiratory collapse (<50% with a sniff or <20% with quiet inspiration) and (ii) right atrial area (end-systole) >18 cm^2^ .^2^ The 2022 update to the guidelines added tricuspid annular plane systolic excursion (TAPSE)/systolic pulmonary arterial pressure (sPAP) ratio <0.55 mm/mmHg as a category A sign of PH.^2^ As this study was conducted prior to the 2022 update of the guidelines, TAPSE/sPAP ratio was not measured.

**Supplemental Appendix 2**. Evaluation of the contribution of each of the echocardiographic parameters recommended by the 2015 European Society of Cardiology (ESC)/European Respiratory Society (ERS) guidelines for determining probability of pulmonary hypertension (PH).

For each parameter, the number of patients who had (i) an abnormal reading, (ii) a normal reading or (iii) no information on that parameter was reported, together with the prevalence of right heart catheterization (RHC)-confirmed PH among these groups. We also report the likelihood ratios (LR+) for these parameters; i.e. the probability that a patient with PH had an abnormal reading compared with a non-PH patient. Logistic regression was used to identify which of the seven echocardiographic parameters contributed the most to distinguishing PH from non-PH. The parameters were included in the model after categorization (normal *versus* abnormal), as there were too many missing values to run regression models using variables in their continuous form. Results are shown for when missing values are combined either with normal or with abnormal values.

**Supplemental Appendix 3**. Statistical analysis.

Continuous variables were summarized using means and standard deviation (SD) and categorical data were summarized with number and frequency. Sensitivity and specificity were computed and presented together with 95% two-sided Wilson score confidence intervals.

The Wald chi-square test statistic was used for the logistic regression analyses to determine which of the seven echocardiographic parameters were statistically significant (p<0.05 two-sided) contributors to the model. All analyses were performed using SAS v9.4 (SAS Institute, Cary, NC, USA).

**References**

1. Galiè N, Humbert M, Vachiery J-L, et al. 2015 ESC/ERS Guidelines for the diagnosis and treatment of pulmonary hypertension. The Joint Task Force for the Diagnosis and Treatment of Pulmonary Hypertension of the European Society of Cardiology (ESC) and the European Respiratory Society (ERS): Endorsed by: Association for European Paediatric and Congenital Cardiology (AEPC), International Society for Heart and Lung Transplantation (ISHLT). *Eur Respir J.* 2015;46:903-975.

2. Humbert M, Kovacs G, Hoeper MM, et al. 2022 ESC/ERS Guidelines for the diagnosis and treatment of pulmonary hypertension: Developed by the task force for the diagnosis and treatment of pulmonary hypertension of the European Society of Cardiology (ESC) and the European Respiratory Society (ERS). Endorsed by the International Society for Heart and Lung Transplantation (ISHLT) and the European Reference Network on rare respiratory diseases (ERN-LUNG). *Eur Heart J.* 2022;43:3618-731.
